# Treatment of Steroid-Resistant Nodular Episcleritis With Tacrolimus: A Case Report

**DOI:** 10.7759/cureus.47057

**Published:** 2023-10-15

**Authors:** Ali Hendi Alghamdi

**Affiliations:** 1 Ophthalmology, Faculty of Medicine, Al Baha University, Al Aqiq, SAU

**Keywords:** nsaids, tacrolimus, steroids, nodular episcleritis, episclera

## Abstract

A 46-year-old male, with no chronic medical illness, complained of pain, tearing, and redness for one-month duration, with no photophobia, discharge, or decrease in visual acuity. Examination revealed a small, painful, red swelling in the left sclera. Slit-lamp examination using a narrow bright slit beam revealed edema of the episcleral layer and injection of the superficial episcleral blood vessels. The rest of the anterior segment exam and fundoscopy were normal. The laboratory investigations and systemic workup were normal. The patient was initially treated with prednisolone acetate (Pred Forte) 1% every three hours per day for one week, and then four times per day for another week, and tapered gradually over eight weeks with systemic nonsteroidal anti-inflammatory drugs (NSAIDs) as diclofenac sodium for eight weeks with mild improvement of clinical symptoms, but the size of the lesion remained without any change and the patient started to have a relapse of symptoms at the end of the course. Topical tacrolimus drops of 0.1% concentration were prepared in the pharmacy under complete sterile precautions and were used four times per day for the following six weeks duration instead of the initial therapy (steroids and NSAIDs). Tacrolimus drops were then tapered gradually over another six weeks duration. The patient showed dramatic suppression of inflammation and exceptional remission of symptoms with complete resolution of the episcleritis. Topical tacrolimus is very effective in the treatment of nodular episcleritis, which is resistant to steroid therapy. Patients with nodular episcleritis suffer from prolonged bouts of inflammation that are characteristically more painful than the diffuse type and may be associated with other systemic diseases. The case is steroid-resistant nodular episcleritis, which did not respond to the usual treatment and showed a good response to treatment with tacrolimus, which was first introduced in episcleritis. Tacrolimus is being used in other ocular diseases, but its use in episcleritis is unique.

## Introduction

The episclera is a thin outermost layer of loose connective tissue between the conjunctiva and sclera that receives blood supply from superficial and deep vascular plexi that are derivatives from the anterior ciliary arteries, which is a branch from the ophthalmic artery. Episcleritis is defined by the sudden onset of inflammation in the episclera and superficial episcleral vascular plexi [1,2]. The cardinal features include irritation, redness, epiphora, and watering of the eye with preservation of vision [3,4].

Episcleritis is also known as phlegmatous conjunctivitis, subconjunctivitis, and episcleritis periodica fugax. The majority of patients respond quickly to topical therapy and do not alter the acuity of vision. Furthermore, some patients required no treatment and the condition improved completely over a short course [5]. A small percentage of patients have an underlying systemic disease that requires further workup and management [6].

Most episcleritis cases have no definite cause. Some cases may be idiopathic but may be associated with infection, autoimmune (30%) as Crohn's disease, rheumatoid arthritis, psoriatic arthritis, ulcerative colitis, and other diseases, or may be associated with collagen vascular disorders such as polyarteritis nodosa and granulomatosis with polyangiitis [6,7]. Some reported cases of episcleritis have a seasonal variation and more recurrence rate in the spring or fall [7]. Some patients experience exposure to some predisposing factors such as allergy, stress, hormonal changes, and trauma [7,8].

The pathophysiology of episcleritis is inadequately understood [9]. Histopathological examination revealed that the inflammatory reaction is restricted only to the superficial episcleral vascular network and the presence of non-granulomatous inflammation, along with vasodilatation and perivascular mononuclear infiltrates [10].

Episcleritis may be unilateral or bilateral. Two different clinical patterns, i.e., simple/diffuse, sectoral, or focal/nodular, were recognized. The most common type is the diffuse type, which is characterized by recurrent sessions of inflammation ranging from moderate to severe lasting for 10 days, and may be prolonged as in cases associated with systemic diseases. The diffuse type usually resolves within two to three weeks with one to three-month recurrent intervals [11]. Nodular episcleritis implies a localized process with a well-defined elevated area [12].

Patients with nodular, sectoral, or focal episcleritis revealed prolonged bouts of inflammation that are characteristically more painful than the diffuse type. Those patients are usually linked with systemic disorders [13].

## Case presentation

A 46-year-old male, with no chronic medical illness, complained of pain and tearing redness over a one-month history, with no photophobia, discharge, or decrease in visual acuity. The examination revealed a small painful red swelling in the left sclera. The patient was on Maxitrol drops four times per day and ointment at bedtime for one month with minimal improvement. The diagnosis of nodular scleritis was made.

**Figure 1:**
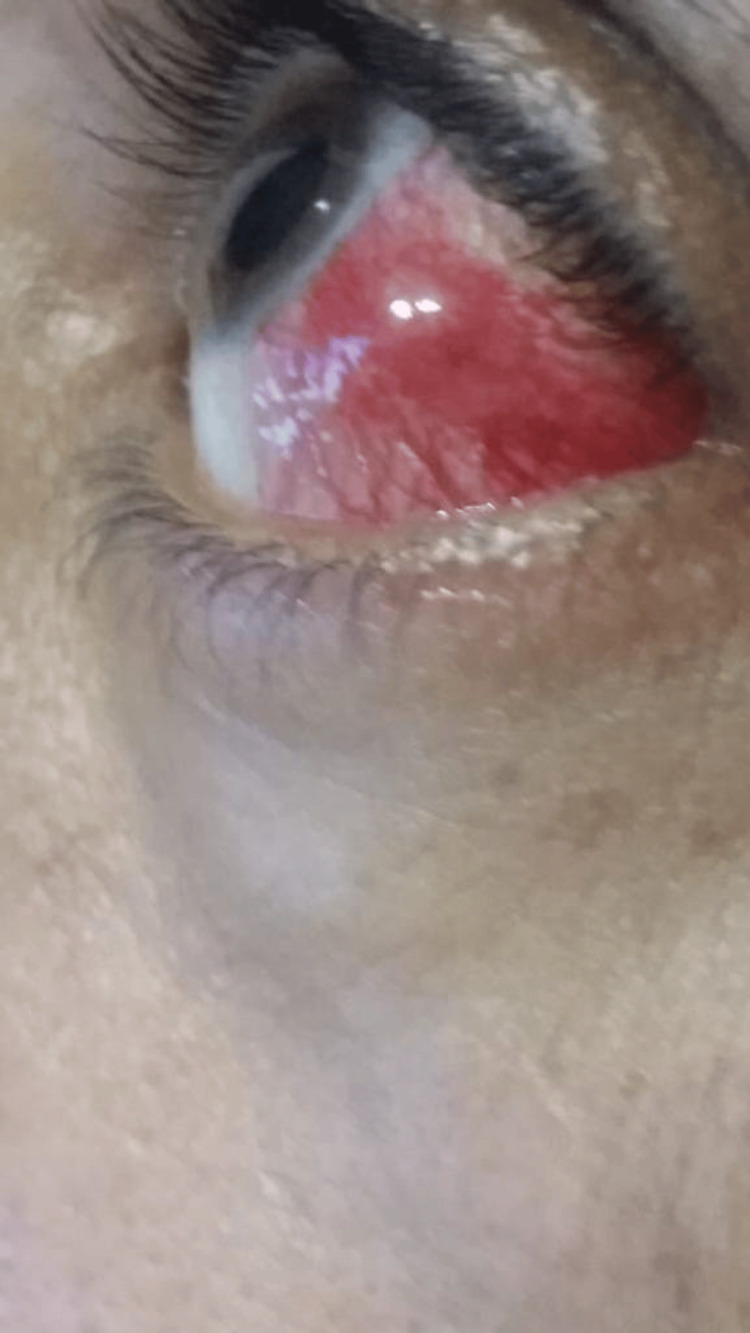
A photograph of the patient with nodular episcleritis in the initial presentation with steroid treatment stage showing a deep-red episcleral mass in the left eye.

Slit-lamp examination using a narrow bright slit beam revealed edema of the episcleral layer and injection of the superficial episcleral blood vessels. To differentiate between episcleritis and scleritis, we determined the depth of the congested vessels by instilling one drop of 2.5% phenylephrine solution in the left eye, and the vasculature was evaluated after 15 minutes and revealed a white eye with blanching of the blood vessels. This test confirmed the diagnosis of episcleritis. On examination, a stable, smooth “deep red” colored mass measuring 8 mm × 5 mm was located in the left eye and expanded close to the lateral canthus. The mass was free from the conjunctiva and all the vessels were lifted by it (Figure 1).

Fundus examination was normal, and the intraocular pressure was normal without limitation of extraocular muscle movements in the left eye. The rest of the anterior and posterior segments were normal on slit lamp examination. To evaluate the patient against infections and autoimmune disorders, investigations were done. These investigations included complete blood count (CBC) and the obtained results for CBC are presented in Table 1.

**Table 1:** A table showing the results of CBC of the patient with nodular episcleritis of the present case. CBC: complete blood count; RBCs: red blood cells; HB: hemoglobin; HCT: hematocrit; WBCs: white blood cells; RDW: red cell distribution width; MCV: mean corpuscular volume; MCH: mean corpuscular hemoglobin; MCHC: mean corpuscular hemoglobin concentration; MPV: mean platelet volume.

Parameters	Results	Reference range
RBCs	4.6/cmm	4.5-5.5/cmm
HB	13.9 g/dL	13.8-15.2 g/dL
HCT	45%	45-55%
WBCs	7400/cmm	7000-11,000/cmm
Platelets	240,000/cmm	240,000-450,000/cmm
RDW	11	11-15
MCV	83	80-100 fL
MCH	28	27-32 pg
MCHC	34%	32%-36%
MPV	10 fL	6-12 fL

Further investigations have been conducted such as C-reactive protein, erythrocyte sedimentation rate (ESR) rheumatoid factor (Rh), anti-cyclic citrullinated peptide (ACCP), anti-nuclear antibody (ANA), human leukocyte antigen-B27 (HLA-B27), anti-neutrophil cytoplasmic antibody (ANCA), fluorescent treponemal antibody absorption (FTA-ABS) test, and Venereal Disease Research Laboratory (VDRL) test. In addition, IgM and IgG for TORCH (Toxoplasma, rubella, cytomegalovirus, herpes virus) were negative. All these results are presented in Table 2.

**Table 2:** A table showing the laboratory results of the patient with nodular episcleritis in the present case.

Test	Results	Reference range	Interpretation
C-reactive protein	3 mg/L	Less than 6 mg/L	Normal range
Erythrocyte sedimentation rate (ESR)	8 mm/hr	1-13 mm/hr	Normal range
Rheumatoid factor (Rh)	10 IU/mL	Less than 15	Normal range
Anti-cyclic citrullinated peptide (ACCP)	12 U/mL	Less than 20 U/mL	Normal range
Anti-nuclear antibody (ANA) by enzyme-linked immunosorbent assay (ELISA)	Negative	Negative	Normal
Human leukocyte antigen-B27 (HLA-B27)	Negative	Negative	Normal
Anti-neutrophil cytoplasmic antibody (ANCA) by ELISA	0.03/mL	0.00-0.22 u/mL	Normal
Fluorescent treponemal antibody absorption (FTA-ABS) test	Nonreactive	Nonreactive	Normal
Venereal Disease Research Laboratory (VDRL) test	Negative	Negative	Normal
TORCH (Toxoplasma, rubella, cytomegalovirus, herpes virus) screen IgM, IgG	Negative	Negative	Normal

Other laboratory investigations such as uric acid, serum creatinine, and urine analysis also indicated no abnormality. The results of these investigations were of normal range with no abnormality obtained except for mild normocytic hypochromic anemia. A two-dimensional image optical coherence tomography (OCT-B) was also done for the patient and showed no abnormalities.

**Figure 2:**
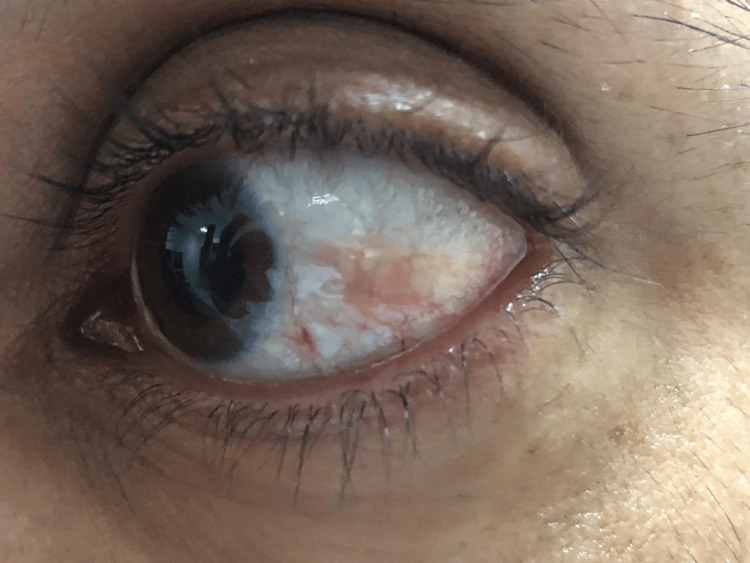
A photograph of the patient with nodular episcleritis two months after treatment with tacrolimus eye drops 0.1% demonstrating marked improvement of the nodular episcleritis.

The management protocol was started with prednisolone acetate (Pred Forte) 1% every three hours for one week followed by four times per day for another week and tapered gradually over eight weeks in addition to nonsteroidal anti-inflammatory drugs (NSAIDs) such as diclofenac sodium (Voltaren tablet 50 mg twice per day) with mild improvement. The lesion remained the same after tapering the steroid to once daily over one month and the symptoms returned, and the patient became dissatisfied. Topical tacrolimus drops of 0.1% concentration were prepared in the pharmacy under complete aseptic precautions and were used four times per day for six weeks duration instead of the initial therapy (steroids and NSAIDs). Tacrolimus drops were then tapered gradually over another six weeks duration. The case showed dramatic suppression of inflammation and subsequent exceptional remission of the patient's symptoms with complete resolution of episcleritis (Figure 2).

**Figure 3:**
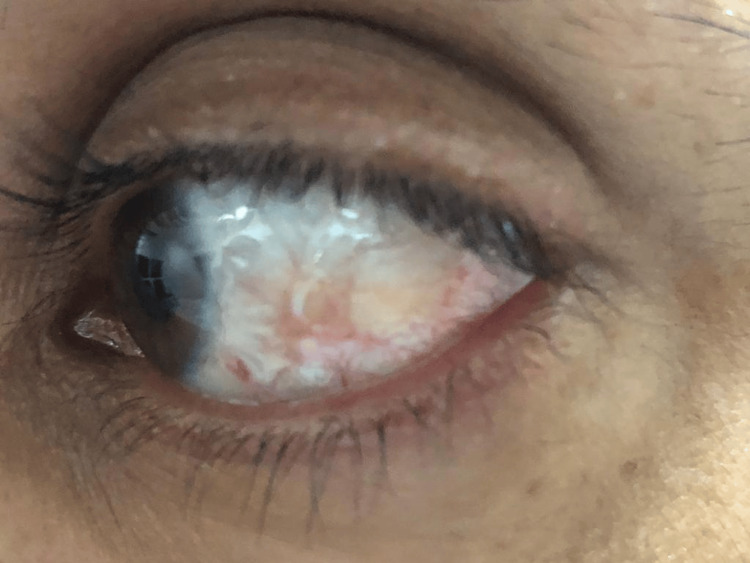
A post-treatment photograph of the patient with nodular episcleritis at a six-month follow-up demonstrating marked improvement of the nodular episcleritis.

**Figure 4:**
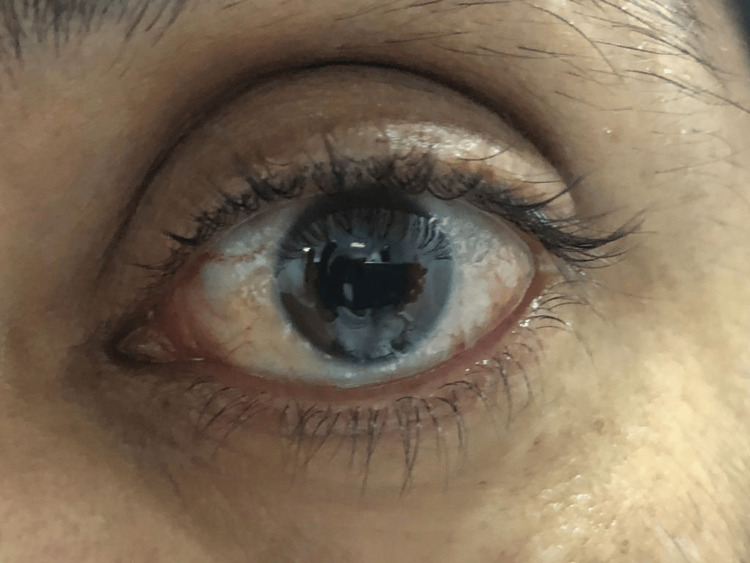
A post-treatment photograph of the left eye at 12-month follow-up demonstrating quite normal eye with no evidence of recurrence.

Follow-up of the patient at a six-month interval (Figure 3) and after one year revealed no recurrence and no abnormalities were found. The patient was happy with the results of the treatment with no significant side effects (Figure 4).

## Discussion

Nodular episcleritis accounts for 15-30% of cases of episcleritis and is mostly unilateral in about 80% [12], as presented in the current case. In general, episcleritis is frequently a self-limiting disease with an approximately 15-20% resolution rate if not treated. Topical lubricants and patient education may be sufficient [13]. Many ophthalmologists start treatment with a topical steroid and increase the dose if the inflammation persists or switch to a more effective topical steroid and oral NSAIDs [14]. This was initially tried in the management of our presenting case.

The treatment of nodular episcleritis is one of the great challenges for ophthalmologists due to the variety of clinical manifestations and outcomes that are sometimes dissatisfactory with a high failure rate [15,16]. Episcleritis is generally benign, but some articles reported a few complications, especially with recurrent disease. These involve anterior and intermediate uveitis, and declining acuity of vision, which is characteristically credited to progressing cataracts and glaucoma [16]. Cataract and glaucoma development are closely related to steroid treatment as a part of episcleritis management.

In the present case, we used topical tacrolimus drops at 0.1% concentration after topical steroid therapy for managing the nodular episcleritis and showed dramatic suppression of inflammation and subsequent exceptional remission of patients’ symptoms and complete resolution. The idea for using tacrolimus in nodular episcleritis comes from its potent anti-inflammatory effect in addition to its uses in different ocular diseases. This is consistent with observations that these diseases have nearly similar predisposing and risk factors with nodular episcleritis as well as the pathogenesis of each with some differences [17,18].

As reported, previous studies showed that episcleritis may be caused by allergic conditions, and using tacrolimus may have beneficial effects in treating those conditions, use of tacrolimus was decided at a dose of 0.1% [19], which showed a dramatic response and the case improved completely with no recurrence after one-year follow-up. The only side effect that appeared was the immediate burning sensation that improved gradually. Tacrolimus alleviates the process of active inflammation, prevents flare-ups, and reduces the elevation of intraocular pressure that is seen in patients who receive steroids. Previous research and workup described side effects of tacrolimus, involving a burning sensation usually after application [20-22], Kaposi varicelliform eruption [23], activation of herpes simplex dendritic keratitis [24], and molluscum contagiosum development [25].

Tacrolimus is a calcineurin inhibitor, an immunosuppressive drug, that acts by inhibiting the activation of the transcription nuclear factor of activated T cells (NFAT) [26]. NFAT transcription factors control broad processes in the T cells that define the functions and fate of different T cell populations. The ability of NFAT to control diverse programs of gene expression depends on its capability to collaborate with several transcription factors and, consequently, participate in calcium signaling, which facilitates NFAT activation and traveling from cytosol to the nucleus and so interleukins (ILs) transcription occurs, especially IL-2. Therefore, inhibition of activation of the NFAT pathway by tacrolimus blocks the induction of transcription of a specific gene and so prevents the IL-2 synthesis [27], which is necessary for the proliferation of T-lymphocyte, and therefore, diminishes the release of interferon-g and other cytokines such as IL-3, IL-4, and IL-5. Suppression of these cytokines by the effect of tacrolimus damages the synthesis of prostaglandin D2 and reduces histamine release from mast cells [28].

Tacrolimus with a concentration of 0.02-0.1% has been used in varieties of ophthalmologic diseases such as allergic conjunctivitis [19,29], this is partially consistent with the present trial. Because of the hydrophobic character of tacrolimus, it is very effective in the management of inflammatory ocular surface diseases that belong to cell-mediated immune responses [30], this is consistent with the present treatment.

The majority of previous studies established that topical tacrolimus is an alternative effective treatment for ocular refractive allergy with a reduction of the recurrence rate despite the long-term use of antihistamines or steroids [29-38]. Several research works have advocated the opportunity of using topical tacrolimus as an effective substitute to corticosteroids in restricted cases of sclerokeratitis [39-41] but no report described its use in nodular episcleritis. A study revealed results supporting a wider application of topical tacrolimus in the management of patients with well-known refractory inflammatory ocular surface diseases such as necrotizing scleritis, chronic cicatrizing conjunctivitis, and Mooren ulcer. In line with our present finding, tacrolimus attains outstanding remission maintenance for two to 25 months following a gradual tapering of tacrolimus administration [42-44].

## Conclusions

Management of nodular episcleritis is usually simple and most cases respond to steroid therapy; however, some cases do not respond to steroids, or show a mild response with a high recurrence rate. We reported a case of nodular episcleritis treated with tacrolimus drops of 0.1% concentration and the outcome was excellent after two to three months of therapy. Follow-up of the patient was done regularly for up to one year and no recurrence was uneventful.
